# Whole-Exome Sequencing Revealed New Candidate Genes for Human Dilated Cardiomyopathy

**DOI:** 10.3390/diagnostics12102411

**Published:** 2022-10-05

**Authors:** Ylenia D’Agostino, Domenico Palumbo, Maria Rosaria Rusciano, Oriana Strianese, Sonia Amabile, Domenico Di Rosa, Elena De Angelis, Valeria Visco, Fabio Russo, Elena Alexandrova, Annamaria Salvati, Giorgio Giurato, Giovanni Nassa, Roberta Tarallo, Gennaro Galasso, Michele Ciccarelli, Alessandro Weisz, Francesca Rizzo

**Affiliations:** 1 Department of Medicine, Surgery and Dentistry ‘Scuola Medica Salernitana’, University of Salerno, 84081 Baronissi, Italy; 2 Medical Genomics Program, AOU ‘SS. Giovanni di Dio e Ruggi d’Aragona’, University of Salerno, 84131 Salerno, Italy; 3 Clinical Research and Innovation, Clinica Montevergine S.p.A., 83013 Mercogliano, Italy; 4 Genome Research Center for Health, Campus of Medicine, University of Salerno, 84081 Baronissi, Italy; 5 Department of Cardiology and Intensive Care Unit, San Luca Hospital, 84078 Vallo della Lucania, Italy

**Keywords:** cardiovascular disease, dilated cardiomyopathy (DCM), next-generation sequencing (NGS), whole-exome sequencing (WES), variant detection

## Abstract

Dilated cardiomyopathy (DCM) is a complex disease affecting young adults. It is a pathological condition impairing myocardium activity that leads to heart failure and, in the most severe cases, transplantation, which is currently the only possible therapy for the disease. DCM can be attributed to many genetic determinants interacting with environmental factors, resulting in a highly variable phenotype. Due to this complexity, the early identification of causative gene mutations is an important goal to provide a genetic diagnosis, implement pre-symptomatic interventions, and predict prognosis. The advent of next-generation sequencing (NGS) has opened a new path for mutation screening, and exome sequencing provides a promising approach for identifying causal variants in known genes and novel disease-associated candidates. We analyzed the whole-exome sequencing (WES) of 15 patients affected by DCM without overloading (hypertension, valvular, or congenital heart disease) or chronic ischemic conditions. We identified 70 pathogenic or likely pathogenic variants and 1240 variants of uncertain clinical significance. Gene ontology enrichment analysis was performed to assess the potential connections between affected genes and biological or molecular function, identifying genes directly related to extracellular matrix organization, transcellular movement through the solute carrier and ATP-binding cassette transporter, and vitamin B12 metabolism. We found variants in genes implicated to a different extent in cardiac function that may represent new players in the complex genetic scenario of DCM.

## 1. Introduction

Dilated cardiomyopathy (DCM) is considered one of the world’s leading causes of heart failure and transplantation [1]. This clinical condition is defined as a progressive and usually irreversible disease of the myocardial muscle, characterized by left ventricular or biventricular dilatation that consequently determines impaired contractile function [2]. DCM can be caused primarily by genetic and nongenetic factors, such as hypertension, valve disease, myocardial inflammation due to infection, and exposure to toxins [3,4].

The true prevalence of DCM and genetically mediated DCM is not fully known. In the general population, the recently estimated incidence is 1:2500 patients, increasing to 1:250–400 in patients with heart failure [5].

In the past, linkage analyses have made it possible to clarify the pathogenetic mechanisms underlying idiopathic dilated cardiomyopathy and to identify some disease genes with important clinical and therapeutic implications [6].

In patients with familial DCM, approximately 40% of cases are carriers of mutations in causing genes. Furthermore, pathogenic variants can be identified in sporadic DCM, even if the frequency of these variants is not well defined in the population [7,8]. Most genetic DCM is inherited as an autosomal dominant trait with variable expression and penetrance, although some specific forms of mitochondrial, autosomal recessive, and X-linked recessive inheritance have been reported [7,9,10,11]. Moreover, de novo mutations can also contribute to genetic cardiomyopathy [7]. Moreover, genetic predisposition can increase susceptibility to environmental factors, leading to the appearance of highly variable phenotypes. The autosomal dominant form of the disease has been associated with mutations in genes encoding for nuclear membrane, cytoskeletal, sarcomeric, and intercalated disc proteins [12,13]. The etiopathogenesis remains almost indefinite in many cardiomyopathies, with difficulties in the therapeutic approach and the prognostic definition [14]. 

A genetic diagnosis is essential not only to allow prognostication and possibly the personalization of patient management but also for family screening and predicting the risk of recurrence.

Next-generation sequencing (NGS) technologies are effective procedures that can fulfill this gap; however, traditional targeted sequencing panels of clinically relevant genes allow the identification of fewer candidate genes, mainly involved in autosomal dominant forms and with variable penetrance [15]. 

Today, whole-exome sequencing (WES) has the potential to revolutionize the way to practice medicine; however, the utility of WES for the clinical diagnosis, risk stratification, and management of non-inherited DCM has not yet been specifically established [16].

Here, we investigated the whole exome of patients affected by DCM to identify known and possible new genes involved in DCM pathogenesis.

## 2. Materials and Methods

### 2.1. Patients’ Clinical Characteristics

Fifteen volunteer patients aged 56 ± 6 years with a diagnosis of sporadic idiopathic dilated cardiomyopathy (DCM) were enrolled in the heart failure outpatient clinic of the University Hospital “Ruggi d’Aragona” of Salerno, Italy. The study was conducted in accordance with the Declaration of Helsinki and approved by local ethics committees for human research (prot. SCCE n°10650) and all patients signed written informed consent.

Sporadic DCM was diagnosed [1] by the presence of fractional shortening <25% (>2 SD, standard deviation), ejection fraction <45% (>2 SD), or by Magnetic Resonance Imaging (MRI) < 2 SDs below the age- and sex-adjusted average [17] and left ventricular (LV) end-diastolic diameter >117% (>2 SD of the predicted value of 112% corrected for age and body surface area), in the absence of significant coronary artery disease or valvular disease, documented myocarditis, systemic disease, sustained arterial hypertension, or congenital cardiac malformation. The electrocardiograms (ECGs) of these patients showed a sinus rhythm without left bundle branch block and normal limits of T-waves. The heart rates of patients ranged from 60 to 100 bpm and the duration of QRS was <120 ms. When appropriate information was available, DCM cases were excluded if family history was identified in at least one affected relative [9]. The baseline characteristics of patients are listed in Table 1.

### 2.2. Whole-Exome Sequencing (WES)

WES was performed on the fifteen affected patients and analyzed for research purposes. Genomic DNA (gDNA) was isolated from blood cells using the automated QIAsymphony system (QIAGEN); quality control checks were performed using the Qubit dsDNA BR Assay Kit (ThermoFisher Scientific, Waltham, MA, USA) on a Qubit 2.0 Fluorometer (ThermoFisher Scientific, Waltham, MA, USA) and the Agilent 2200 TapeStation System (Agilent Technologies, Santa Clara, CA, USA) using the DNA genomic assay for the evaluation of gDNA concentration and DIN (DNA Integrity Number), respectively. 

Exome sequencing was performed on 50 ng of gDNA using the SureSelect QXT target enrichment protocol for Illumina Paired-End Multiplexed Sequencing Library (Agilent Technologies, Santa Clara, CA, USA) according to the manufacturer’s instructions. Exome capture was performed using the all-exome human V7, 42 Mb capture kit from Agilent. The resulting libraries were sequenced on NextSeq-500 System (Illumina, San Diego, CA, USA) in a 2 × 150 paired-end format at a final loading concentration of 1.8 pM (Supplementary Table S1).

### 2.3. Bioinformatics Analysis

Paired-end demultiplexed reads were produced using bcl2fastq, and the quality control was performed using FastQC. The reads were trimmed from adapters using Trimmomatic [18] with a minimum length of 30 bp cut-off and a leading and trailing of 10 bp. The resulting reads were mapped on the human genome (hg38) using BWA-MEM (0.7.17). Sorting and indexing were performed using SAMtools (1.10) [19], while the BAM correction and the metrics collections were obtained using Picard (2.20.3). Mutect2 from GATK (4.1.2.0) [20] was used for the variant calling using the af-only-gnomad.hg38.vcf as a germline resource. The filtered variants were annotated using wANNOVAR [21] and VarSome [22]; the database was accessed from 1 April 2022 to 31 May 2022. All calculations and statistical analyses were performed in R (version 4.0.2–released under the “GNU Public License” GPL-2|GPL-3).

The obtained data were first filtered for genomic variations in genes previously associated with DCM (CUI: C0007193). Specifically, we used a list of 512 genes reported in the DisGeNET database (https://www.disgenet.org/, accessed on 7 July 2021) for inherited cardiomyopathy; afterward, genomic variants in all the remaining genes were considered. The list of genes containing variants classified by Varsome as pathogenic, likely pathogenic, and of uncertain significance (but excluding the 512 genes already considered as associated with DCM) was imported into WebGestalt (http://www.webgestalt.org, accessed on 4 March 2022) [23] and analyzed for over-representation, including the KEGG (Kyoto Encyclopedia of Genes and Genomes, http://www.kegg.jp/, accessed on 4 March 2022), Reactome (https://reactome.org, accessed on 4 March 2022), and WikiPathways (https://wikipathways.org, accessed on 4 March 2022) databases. Significantly enriched terms were identified using a *p*-value significance of *p* < 0.05. 

## 3. Results

### 3.1. Whole-Exome Sequencing of Sporadic DCM Patients Revealed Variants in Genes Known for Their Association with the Disease

In the current study, a cohort of fifteen patients with the clinical and echocardiographic diagnosis of non-familial (sporadic) DCM was enrolled in the University Hospital of Salerno cardiology clinic and studied by WES on peripheral blood DNA.

**Figure 1:**
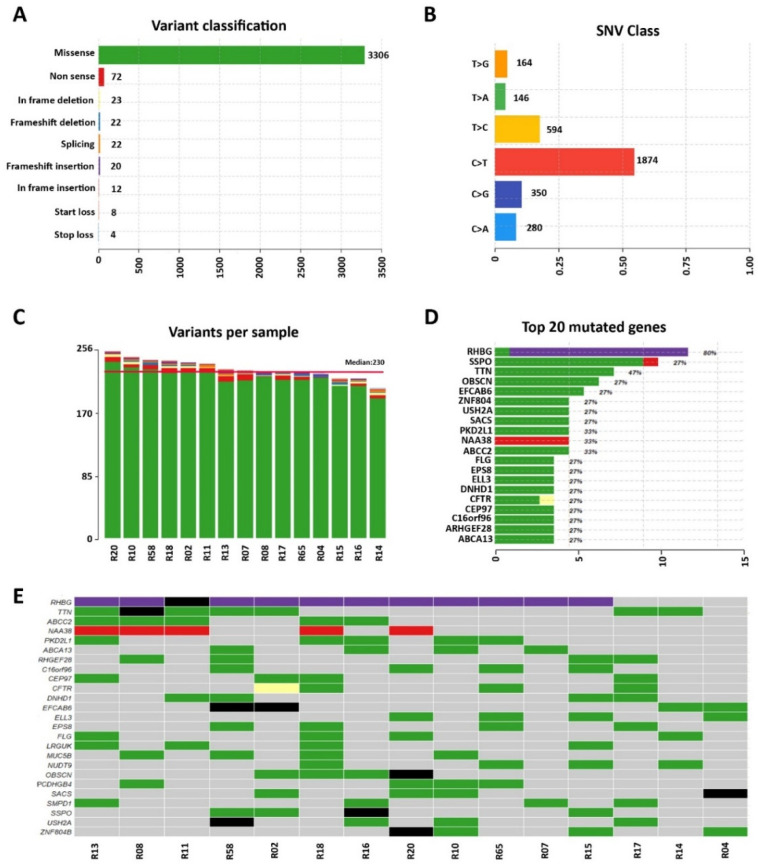
Summary of variants identified in 15 DCM patients. (**A**) The number of variants is subdivided by Human Genome Variation Society (HGVS) classification criteria. (**B**) Single Nucleotide Variant (SNV) classes. (**C**) The number of variants identified per sample. (**D**) Top 20 most frequently mutated genes across the cohort. The percentage on top of the bar represents the number of mutated samples and the bar color indicates the variant type. (**E**) Plot generated by Maftools with the details of mutations found in each patient in the top 25 mutated genes sorted and ordered by decreasing frequency. The different variant types are defined by colored boxes: green for missense variants; purple for a frame-shift variant; yellow for in-frame variants; red for non-sense variants; and black for multiple variants detected in a specific gene.

The clinical characteristics of the probands are summarized in Table 1. The mean ± SD age of the participants was 56 ± 6 years at diagnosis and the cohort comprised 67% males (n = 10). We generated high-coverage WES obtaining ~80 million reads/sample, a mean coverage of 135X, and 98% of 10X covered target regions (Supplementary Table S1). The mutational signature was defined using state-of-the-art bioinformatics methods. We removed the variants under the following criteria for prioritizing the relevant ones: (a) sequencing depth < 10; (b) UTR (untranslated region), synonymous, intronic variants (except the ones considered as spliced variants and located at exon-intron junctions ranging from −5 to +5); (c) allele frequency (AF) < 0.3; and (d) minor allele frequency (MAF) >0.01 from 1000 genomes consortium, ExAC, and GnomAD database. After filtering, 3489 variants were obtained, among which 3306 (94.7%) were missense mutations, 72 (2.1%) non-sense mutations, 22 (0.6%) frameshift deletions, 23 (0.6%) in-frame deletions, 22 (0.6%) splicing mutations, 20 (0.6%) frameshift insertions, 12 (0.3%) in-frame insertions, 8 (0.2%) translational start site loss, and 4 (0.1%) stop loss mutations (Figure 1A). We found 3408 single nucleotide substitutions, with C->T (n = 1874) dominating the mutation spectrum in the 15 DCM samples (Figure 1B). Each selected patient carried an average of 230 variants (Figure 1C). 

RHBG was among the 20 most frequently mutated genes, altered in 80% of patients (Figure 1D,E). Twelve out of fifteen patients present a frameshift insertion (rs11303415), for which no clinical significance is reported in the ClinVar database but is considered as of uncertain significance by the American College of Medical Genetics and Genomics (ACMG) classification (VarSome); one patient has a missense variant (rs200320178) also thought of as of uncertain significance. 

Among the others, we also identified 11 variants (10 classified as benign/likely benign and 1 as of uncertain significance) in the SSPO (SCO-Spondin) gene (Figure 1D,E), encoding for a secreted glycoprotein involved in central nervous system development and the modulation of neuronal aggregation [24]. Eight missense variants have been detected in the TTN gene that codifies for Titin, an integral sarcomeric protein involved in passive force transmission that plays essential roles in sarcomere organization, elasticity, and cell signaling [25]. All the variants reported in this gene were classified as benign by VarSome.

Seven variants, classified as likely benign, have been identified in the OBSCN gene that codifies for Obscurin. This giant cytoskeletal protein plays critical structural and regulatory roles in striated muscles [26]. It has been reported that the N-terminus of Obscurin binds to the most C-terminal domain of Titin. Interestingly, structural defects in both of these proteins might have pathogenic implications for hereditary myopathies [27].

Six missense variants, classified as benign by VarSome, have been detected in the EFCAB6 gene. This gene emerged as a new cardiac marker for cardiac hypertrophy from the transcriptomic analysis of rat hearts [28].

Five missense variants, classified as benign (3/5), likely benign (1/5), and of uncertain significance (1/5), have been identified in the USH2A gene encoding for Usherin protein (Figure 1D,E), involved in the maintenance of the periciliary membrane. Mutations within this gene have been associated with Usher syndrome type IIa and retinitis pigmentosa [29].

Since no common variants showed a direct correlation with DCM, bioinformatics in silico filter was applied to restrict the analysis on variants affecting 512 known genes, previously associated with dilated cardiomyopathy and reported in the DisGeNET database (https://www.disgenet.org/, accessed on 7 July 2021). 

This analysis identified 102 genomic variants in 68 of 512 selected genes (Supplementary Table S2). All variants were manually curated and classified following Human Gene Mutation Database (HGMD) guidelines, finding 50/102 benign variants, 33/102 likely benign variants, 18/102 variants of uncertain clinical significance (VUS), and 1/102 pathogenic variants in a splicing site of the CD36 gene, coding for a glycoprotein of the platelet surface. 

Among the variants of uncertain significance, six showed high pathogenicity scores (considering more than 10 out 20 prediction algorithms considered) in DOLK, ERBB2, HSP90AA1, MME, and POSTN genes (in 2/15 patients) (Supplementary Table S2). 

### 3.2. Identification of Variants in Genes Not Previously Associated with DCM

**Figure 2:**
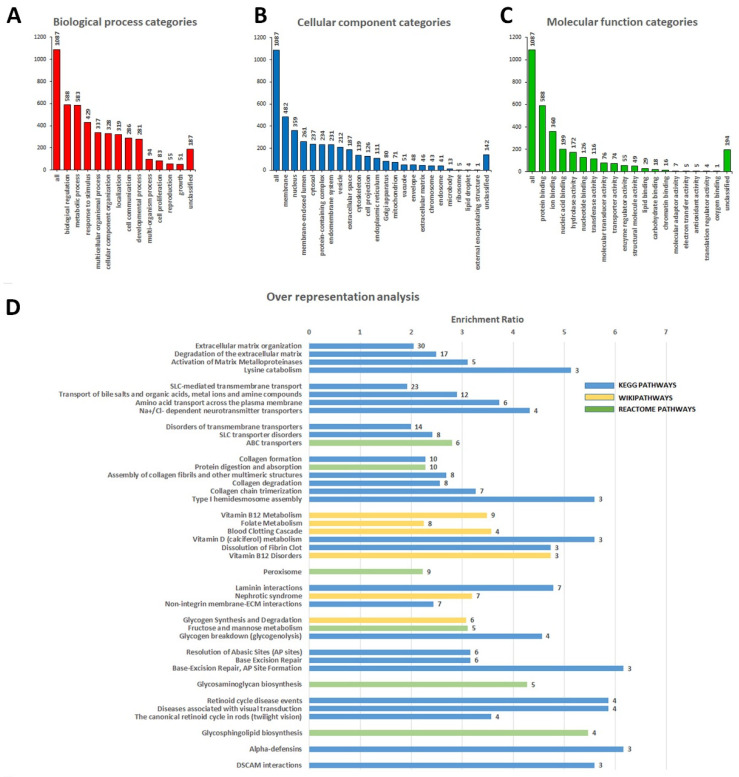
Over-representation analysis (ORA) made with Web-based Gene Set Analysis Toolkit (WebGestalt) using genes containing variants not previously associated with DCM. GO summary showing biological process (**A**), cellular component (**B**), and molecular function (**C**) categories in the red, blue, and green bars, respectively. The height of the bar represents the number of IDs in the user list and the GO category. (**D**) Bar chart of enrichment ratios for KEGG, Reactome, and Wikipathways categories. Enrichment ratio = the number of observed divided by the number of expected genes from each category in the gene list (according to the WebGestalt tool).

Additionally, we also found 19 pathogenic, 50 likely pathogenic variants, and 1222 VUS in our cohort that fall into 1117 genes not previously associated with DCM (Supplementary Table S3). To gain insight into new possible genes that can be related to the DCM phenotype, we performed a gene ontology (GO) term enrichment analysis considering the selected genes. In Figure 2, the relationship between affected genes with biological process categories (A), cellular components categories (B), and molecular functions categories (C) are reported. As can be noted, the biological process analysis shows that most of the genes were engaged in “biological regulation”, “metabolic process”, and “response to stimulus”. Regarding the cellular component, GO terms including “membrane”, “nucleus”, and “membrane-enclosed lumen” resulted in the most relevant ones. Finally, for the molecular function categories, “protein binding”, “ion binding”, and “nucleic acid binding” captured the principal functions. 

Moreover, to further obtain functional insight into DCM pathogenesis and identify new gene sets that could be associated with the disease, we conducted an over-representation analysis (ORA) on the 1117 genes (Figure 2D, Supplementary Table S4A). We obtained different overlaps in genes involved in macro-categories such as extracellular matrix organization, SLC-mediated transmembrane transport, disorders of transmembrane transporters, collagen formation, vitamin B12 metabolism, peroxisome, laminin interaction, glycogen synthesis and degradation, base-excision repair, glycosaminoglycan biosynthesis, retinoid cycle disease events, glycosphingolipid biosynthesis, alpha-defensins, and DSCAM interactions.

According to this analysis, the relevant top-ranked gene set was “extracellular matrix organization” and included the genes: ADAMTS8, CAPN13, CMA1, COL12A1, COL14A1, COL15A1, COL16A1, COL18A1, COL21A1, COL4A5, DST, ICAM1, ITGB4, ITGB6, KLK2, LAMA1, LAMB2, LAMC3, LTBP3, MATN1, MMP24, NID1, OPTC, PHYKPL, PLEC, PLG, SCUBE3, TMPRSS6, TNC, and TTR.

Except for KLK2, MATN1, MME, OPTC, PLG, and TMPRSS6, all the previous genes are highly expressed in heart tissue (based on The Human Protein Atlas database, https://www.proteinatlas.org/, accessed on 4 March 2022). Moreover, information on knockout mouse models is available for 27 out of 30 genes in the MGI database (Mouse Genome Informatics database, http://www.informatics.jax.org/, accessed on 4 March 2022). Among these, 12 present a phenotype directly related to the cardiovascular system (Supplementary Table S4B).

Most of these genes are also shared by “collagen formation” and “laminin interaction” terms. Evidence from the literature reported a pivotal role of extracellular matrix (ECM) components in DCM since fibrosis and consequent ECM remodeling are primary factors for pathogenesis development [30].

Significant enrichment has also been detected for genes related to ATP-binding cassette (ABC) and solute carrier (SLC) transporters, which are two superfamilies with a pivotal role in the pathogenesis of cardiovascular diseases [31].

Finally, ORA analysis also evidenced relevant, significantly enriched categories connected with vitamin B12 metabolism. 

## 4. Discussion

The American Heart Association (AHA) committee classifies cardiomyopathies into three categories: genetic, mixed (genetic and nongenetic), or nongenetic (acquired) [5]. Genetic DCM shows a complex genetic architecture that has been correlated with over 500 genes (https://www.disgenet.org/, accessed on 7 July 2021).

In this study, we used WES on a cohort of sporadic DCM cases to overcome the limitations of a candidate gene approach, with the dual aim of identifying variants in genes known for their association with DCM and possibly discovering new, unsuspected genetic bases of DCM.

Analyzing the known DCM genes, we identified six patients as carriers of heterozygous variants, classified as pathogenic or VUS in five genes (Supplementary Table S2), four of these (CD36, DOLK ERBB2, POSTN) encoding for proteins expressed in the human heart [32] (https://www.proteinatlas.org, accessed on 4 March 2022). One patient is a carrier of a rare (Frequency in Gnomad Genomes= 0.0000527) heterozygous splicing variant in the CD36 gene. CD36, also known as fatty acid translocase (FAT), is the main protein involved in transporting long-chain fatty acids in cardiomyocytes [33]. Fatty acids provide over 70% of the myocardial tissue’s adenosine triphosphate (ATP). Changes in CD36 levels/function have been implicated in the alteration of myocardial metabolism in the pathophysiology of certain cardiovascular diseases [34]. CD36 deficiency is a common condition in patients with hypertrophic cardiomyopathy but has also been reported in rare cases of DCM [35,36,37]. 

For 2/15 patients, we found missense variants in the POSTN gene. Periostin (POSTN) is an integrin-binding protein, highly expressed in the heart, which promotes collagen fibrogenesis and regulates atrioventricular valve maturation during cardiac development [38]. The enhanced expression of POSTN has also been observed in patients with DCM, where it has a role in myocardial ECM remodeling and fibrosis and is associated with the development of diastolic dysfunction [39]. 

DOLK is a gene expressed ubiquitously in humans and encoding an enzyme responsible for the terminal step of dolichol phosphate synthesis; its mutations have been associated with a broad phenotypic spectrum that includes neurological abnormalities, isolated cardiomyopathy, and multi-organ involvement, which may also include ichthyosis [40]. Lefeber et al. [41] have described different pathogenic mutations in the DOLK gene in 11 young patients with predominantly nonsyndromic DCM and showed that dolichol kinase loss determines abnormal N-glycosylation and a reduction of the O-mannosylation of α-dystroglycan, leading to DCM. In our cohort, we identified one patient as a carrier of a heterozygous variant affecting Tyr360, an amino acid located in the 11th transmembrane region and considered a potential phosphorylation site (based on the PhosphoSitePlus database, https://www.phosphosite.org, accessed on 4 March 2022). 

Interestingly, we found also a patient with a variant in the ERBB2 gene; the mutations (Pro489Leu) lie within the extracellular domain of ErbB2, between the receptor L domain (RLD, domain III) and the growth factor receptor domain (GRRD4, domain IV). The ErbB2 gene codifies for a tyrosine kinase receptor and is well known for its critical role in breast cancer progression and acute lymphoid leukemia [42]. However, ErbB2-deficient mice revealed several parameters of dilated cardiomyopathy, including chamber dilation, decreased contractility, and wall thinning, suggesting a possible role of the ErbB2 pathway in the prevention of DCM [43,44]. Moreover, a direct link between Erbb2 activity and the remodeling of myofibrils has been shown in the zebrafish heart [45].

Moreover, we explored variants in genes that are not directly linked to DCM but may open new perspectives to understanding the pathology onset (Supplementary Table S3).

In 4/15 patients of our cohort, we found variants in the SMPD1 gene, three likely pathogenic and one VUS. Sphingomyelin phosphodiesterase 1 (SMPD1) converts sphingomyelins (SMs) to ceramides [46] and is involved in the regulation of immune cell functions and Niemann–Pick disease, a lysosomal storage disorder characterized by hepatosplenomegaly, jaundice, and cytopenia [47]. The abnormal accumulation of ceramides due to the mutation of SMPD1 plays an essential role in acute myocardial ischemia [48]. In cardiomyocytes, ceramides regulate the activation of the volume-sensitive chloride current that controls cardiac electro-mechanical activity, cell volume, and apoptosis [49] and contribute to lipotoxic cardiomyopathy [50].

One patient analyzed showed a variant of the MEFV gene responsible for Familiar Mediterranean Fever (FMF). FMF is a genetic autoinflammatory disorder, characterized by episodic attacks of peritonitis, pleuritis including pericarditis, arthritis, and fever [51]. Generally, the ECG of patients that display this mutation shows an elevated ST segment and the transient enlargement of the cardiac morphology, while echocardiography analysis shows the presence of pericardial effusion. The chronic inflammatory status and the changes in ECG parameters in these patients could predispose them to DCM. Additionally, in the same patient, we observed two variants in the SNTA1 gene (one likely pathogenic and one VUS). SNTA1–Syntrophin Alpha 1–gene encodes for a member of dystrophin-associated protein complex highly expressed in heart tissue (https://www.proteinatlas.org/, accessed on 4 March 2022). Diseases associated with SNTA1 include Long QT Syndrome. In a study by Ueda et al. [52], it has been demonstrated that SNTA1 also interacts with SCN5A, connecting it to the nNOS–PMCA complex in the heart, and a missense mutation in SNTA1 disrupts the association between PMCA4b and SCN5A, leading to an increased late sodium current (I Na) in both a non-cardiomyocyte heterologous expression system and native cardiomyocytes [52]. Since SCN5A is widely and definitively linked to DCM genetic architecture, and considering that a loss of interaction between SNTA1 and SCN5A could alter the sodium channel availability or biophysical properties, we can hypothesize that SNTA1 might contribute to the SCN5A-mediated phenotypes in DCM. Moreover, autonomic dysfunctions, such as prolonged QT dispersion, were previously observed in some patients affected by FMF [53,54,55].

Two patients are carriers of a rare, likely pathogenic variant (rs186739814, Frequencies in Gnomad Exomes 0.00000422) in the KALRN gene, which encodes for a guanine nucleotide exchange factor (GEF) with ubiquitous cell-signaling roles [56]. It has also been reported that Kalirin plays a pivotal role in the signaling of spine morphogenesis, bone growth, and smooth-muscle-cell morphology [57]. Moreover, KALRN was downregulated in the heart tissue of patients affected by both DCM and ischemic cardiomyopathy [58], and mutations within KALRN have been associated with the promotion of stroke and coronary heart disease [56].

Furthermore, through gene ontology analysis, we have identified a group of 32 affected genes involved in the organization of the extracellular matrix (ECM) (Figure 2 and Supplementary Table S4), in which we identified 33 variants (31 VUS and 2 likely pathogenic) in 14/15 patients. Myocardial fibrosis is a major feature in DCM [59]; indeed, in response to this pathological condition, a series of changes in the interstitial myocardial collagen network occurs [60]. During the reparative response, dead cardiomyocytes are mainly replaced by collagen fibrils [61]. Interestingly, most genes enriching our GO term belong to the collagen protein family (COL4A5, COL12A1, COL14A1, COL15A1, COL16A1, COL18A1, COL21A1). Mice lacking COL15A1 and COL18A1 genes present impaired heart function phenotypes; specifically, Col15a1(-/-) knockout leads to a complex cardiac phenotype and predisposes mice to cardiomyopathy [62]. Furthermore, the ECM architecture of the heart wall is ensured by the balance between protein synthesis and degradation mediated by a family of enzymes secreted by fibroblasts known as matrix metalloproteinases (MMPs) [63]. In our dataset, three different variants with uncertain significance have been identified in genes coding for MMPs (MMP24, ADAMTSL5, ADAMTSL8). ADAMTS8 expression was strongly increased in DCM, while in vitro studies evidenced its role into the activation of cardiac fibroblasts [64].

We have also found 13 variants (two pathogenic, two likely pathogenic, and nine VUS) in genes coding for the ABC family of transporters (ABCA3, ABCA4, ABCA9, ABCB6, ABCC2, CFTR); most of these genes encode for proteins involved in ATPase activity, the transport of inorganic cations/anions and the maintenance of cholesterol homeostasis. Several ABC transporters have been linked to cellular outflow and lipid and cholesterol trafficking [65]; thus, their association with the pathogenesis of cardiovascular diseases is not surprising [66]. For the CFTR gene, we reported two patients as carriers of heterozygous missense variants and one showing the loss of codon 508. Mutations in CFTR cause cystic fibrosis (CF) [67]. Still, several studies demonstrate the presence of heart disease with myocardial dysfunction in patients with CF, affecting systolic and diastolic heart function from an early age [68]. In 2020, Liu and colleagues reported that the deficiency of the CFTR gene leads to cardiac dysplasia during zebrafish embryogenesis and is associated with DCM [69].

Numerous VUS has been identified in 23 genes coding for solute carrier (SLC) transporters (Supplementary Table S3). These transporters are involved in homeostatic functions and the development of several organs, including the heart [70]. The alteration of their tasks or expressions may contribute to heart disease development; therefore, their possible role as therapeutic targets is emerging [31].

Finally, ORA analysis evidenced an enrichment of genes associated with vitamin B12 metabolism/disorders, such as APOB, CD320, CUBN, F2, F7, ICAM1, LRP2, MMACHC, MPO, PLAT, and PLG. A recent case report demonstrated that vitamin B12 deficiency is an uncommon but potentially reversible cause of dilated cardiomyopathy [71]. It should be noted that some genes (i.e., F2, F7, PLAT, and PLG) belonging to this category are also shared by the “Blood Clotting Cascade” and “Dissolution of Fibrin Clot” terms. These genes codify, respectively, for prothrombin protein (also known as coagulation factor II), coagulation factor VII, tissue-type plasminogen activator, and plasminogen protein. This evidence correlates with platelet activation, thrombin activation, and fibrinolytic activity increase in patients with DCM, thus conducting a general activation of the coagulation pathway [72,73].

In summary, in a small but highly selected cohort of patients diagnosed with dilated cardiomyopathy, we found variants in genes implicated to a different extent to cardiac function or pathways that are altered in general. Moreover, we have identified new ontologies of genes, such as the extracellular matrix organization, SLC-mediated transmembrane transport, collagen formation, and vitamin B12 metabolism, which may occur in the complex genetic scenario of DCM. 

Even if the limit of our analysis is the low number of patients, we can speculate a multi-locus pathway, where DCM is a final phenotype resulting from various disease genes or genetic injury pathways. Unfortunately, the genetic complexity and heterogeneity of DCM are far from being understood, and genetic testing using multi-gene panels can be reductive. Our study revealed the necessity to use more broad approaches; in fact, more than a single variant can be identified within an individual, thus explaining some features and the genetic complexity of this disease. Future approaches are needed to validate the pathogenicity of the mutations, considering the high number of variants identified by exome sequencing, and extensive case studies to confirm the results.

## Figures and Tables

**Table 1:** Baseline characteristics of dilated cardiomyopathy patients.

Variables	Value
Age, years (mean ± SD)	56 ± 6
BMI (Kg/m^2^)	30.3 ± 7.6
Male, sex n (%)	10 (66.7)
NYHA class	
I	none
II	6
III	9
IV	none
Medical history, n (%)	
Diabetes mellitus	5 (33.3)
Hypertension	8 (53)
Dyslipidemia	5 (31)
Current smoker n (%)	2 (12.5)
eGFR	76.3 ± 29.8
Hemoglobin g/dL	13.4 ± 1.2
Echocardiography parameters	
EF (%)	37.4 ± 9.2
VTD (mL)	206.8 ± 80
EDV (mL)	135.8 ± 67.8
E/E’	11.4 ± 5.9
TAPSE (mm)	18.4 ± 4.8
RVS’ (cm/s)	11.5 ± 1.6
PASP (mmHg)	28.8 ± 4.5
Medication baseline, n (%)	
ß-blocker	13 (86.7)
ACEi/ARB	7 (46.7)
Aldosterone antagonist	9 (60)
Diuretic	10 (66.7)
Sacubitril + Valsartan	4 (26.6)

The table summarizes the clinical and pathological data of all patients included in the study. The clinical indices described include mean age, body mass index (BMI), sex, New York Heart Association (NYHA) class, medical history, eGFR, hemoglobin, echocardiography parameters, and medication baselines. (ACEi, angiotensin-converting enzyme inhibitor; ARB, angiotensin-receptor blocker; BMI, body mass index; EDV, End-diastolic volume; EF ejection fraction; eGFR, estimated glomerular filtration rate; NYHA, New York Heart Association; PASP, pulmonary artery systolic pressure; RVS, Right ventricle shortening; TAPSE, tricuspid annular plane excursion).

## Data Availability

The data that support the findings of this study are available from the corresponding authors upon reasonable request.

## Supplementary Materials

The following supporting information can be downloaded at: www.mdpi.com/article/10.3390/diagnostics12102411/s1, Table S1. Summary statistics of exome sequencing data obtained from the 15 DCM patients; Table S2. Characteristics of variants identified in 512 genes previously associated with DCM in the 15 patients; Table S3. Characteristics of variants identified in genes not previously associated with DCM in the 15 patients; Table S4. Over-representation analysis on the 1117 genes containing pathogenic, likely pathogenic, or 1222 VUS variants.
